# Evaluation of Proclarix in the diagnostic work‐up of prostate cancer

**DOI:** 10.1002/bco2.293

**Published:** 2023-09-29

**Authors:** Basil Kaufmann, Sharon Fischer, Alcibiade Athanasiou, Noémie Lautenbach, Anja Wittig, Uwe Bieri, Florian A. Schmid, Franz von Stauffenberg, Thomas Scherer, Daniel Eberli, Michael A. Gorin, Ralph Schiess, Cédric Poyet

**Affiliations:** ^1^ Department of Urology University Hospital Zurich Zurich Switzerland; ^2^ Milton and Carroll Petrie Department of Urology Icahn School of Medicine at Mount Sinai New York New York USA; ^3^ Proteomedix AG Zurich Switzerland

**Keywords:** biomarkers, biopsy, cathepsin D, CTSD, Proclarix, prostate cancer, thrombospondin 1, THBS1

## Abstract

**Objectives:**

The use of multiparametric magnetic resonance imaging (mpMRI) has been widely adopted in the diagnostic work‐up for suspicious prostate cancer (PCa) and is recommended in most current guidelines. However, mpMRI lesions are often indeterminate and/or turn out to be false‐positive on prostate biopsy. The aim of this work was to evaluate Proclarix, a biomarker test for the detection of relevant PCa, regarding its diagnostic value in all men before biopsy and in men with indeterminate lesions on mpMRI (PI‐RADS 3) during work‐up for PCa.

**Materials and Methods:**

Men undergoing mpMRI‐targeted and systematic biopsy of the prostate were prospectively enrolled. The Proclarix test was evaluated for the detection accuracy of clinically significant PCa (csPCa) defined as Grade Group ≥ 2 and its association to mpMRI results. Further, Proclarix's performance was also tested when adapted to prostate volume (Proclarix density) and performance compared to PSA density (PSAD).

**Results:**

A total of 150 men with a median age of 65 years and median PSA of 5.8 ng/mL were included in this study. CsPCa was diagnosed in 65 (43%) men. Proclarix was significantly associated with csPCa and higher PI‐RADS score (*p* < 0.001). At the pre‐defined cut‐off of 10%, Proclarix's sensitivity for csPCa was 94%, specificity 19%, negative predictive value 80% and positive predictive value 47%. Proclarix density showed the highest AUC for the detection of csPCa of 0.77 (95%CI: 0.69–0.85) compared to PSA, PSAD and Proclarix alone. Proclarix was able to identify all six csPCa in men with PI‐RADS 3 lesions (*n* = 28), whereas PSAD missed two out of six. At optimized cut‐offs, Proclarix density outperformed PSAD by potentially avoiding 41% of unnecessary biopsies.

**Conclusion:**

Proclarix demonstrates high sensitivity in detecting csPCa but may still result in unnecessary biopsies. However, Proclarix density was able to outperform PSAD and Proclarix and was found to be useful in men with PI‐RADS 3 findings by safely avoiding unnecessary biopsies without missing csPCa.

## 1 INTRODUCTION

In recent years, the diagnostic work‐up for prostate cancer (PCa) has emerged to incorporate the routine use of multiparametric magnetic resonance imaging (mpMRI) in men with an elevated prostate specific antigen (PSA) level.^1^ Although it is clear that men with a PI‐RADS 4 or 5 lesion require a diagnostic prostate biopsy, it remains controversial as to whether patients with a grade 3 lesion require the same.^2^, ^3^ This is because only 4%–27% of men with a PI‐RADS 3 lesion will ultimately be found to have clinically significant PCa (csPCa).^3^, ^4^, ^5^ In contrast, 22%–57% and 72%–79% of men with a PI‐RADS 4 or 5 lesion will be found to have csPCa, respectively.^6^, ^7^

Considering the high infectious complication rate from prostate biopsy^8^ and the potentially negative downstream consequences of needlessly diagnosing low grade PCa,^9^ it is of critical importance to more accurately risk stratify patients with an equivocal finding on mpMRI prior to moving forward with a biopsy. Although prior work has shown that the incorporation of PSA density (PSAD) into the decision to perform a prostate biopsy in men with a PI‐RADS 3 lesion on mpMRI improves the diagnostic yield for csPCa,^10^, ^11^ the discriminatory ability of this parameter alone still leads to a significant number of unnecessary prostate biopsies.^12^

Thus, additional markers are needed to further estimate the risk of csPCa in men with a PI‐RADS 3 lesion on mpMRI.

Proclarix (Proteomedix AG, Switzerland) is a novel serum‐based assay that was developed to aid in the risk stratification of men with an elevated PSA level.^13^ This test, which incorporates serum total PSA (tPSA), free PSA (fPSA), cathepsin D (CTSD), thrombospondin 1 (THBS1) and patient age,^14^ has been shown to detect csPCa with more favourable diagnostic performance characteristics as compared to percent fPSA (%fPSA) and the ERSPC risk calculator.^15^, ^16^, ^17^, ^18^, ^19^, ^20^, ^21^, ^22^ One limitation of the available literature on the Proclarix test is that all papers relied upon the results of a 10–14 core systematic biopsy of the prostate with or without targeted biopsy core.^15^, ^18^, ^19^, ^20^ Although this practice is typical in the prostate cancer biomarker literature, concerns remain regarding the accuracy of this somewhat limited method of sampling the prostate.^6^, ^23^ For example, at the time of radical prostatectomy, up to 30% of patients will be upgraded or downgraded following a 12–14 core biopsy.^24^ In light of this, it is possible that prior analyses of the Proclarix test were impacted by sampling bias.

In this study, we aimed to compare the performance of the Proclarix test to standard clinical parameters in a contemporary biopsy cohort of men who underwent an mpMRI followed by a transperineal saturation biopsy of the prostate. Importantly, we perform a subset analysis of the diagnostic yield of this test in patients with an indeterminate mpMRI result.

## 2 PATIENTS AND METHODS

### 2.1 Study design

This is a single‐centre, prospective study designed to evaluate the diagnostic performance of the Proclarix test for diagnosing csPCa (grade group ≥ 2 PCa) among men undergoing a combined systematic and targeted prostate biopsy. The study is in line with the STARD 2015 reporting guidelines^25^ and was approved by the local ethics committee (KEK Nr. 2016‐00075). All participants of the study provided written informed consent.

### 2.2 Participants and setting

The study involved 150 men who underwent a prostate biopsy at the University Hospital of Zurich between January 2019 and March 2022. Patients were referred for a prostate biopsy on the basis of an elevated or rising PSA level. Findings on digital rectal exam and family history of prostate cancer also informed the decision as to whether to perform a prostate biopsy. Additionally, this decision took into account patient life expectancy and concomitant illnesses.

Before undergoing a biopsy, each patient underwent mpMRI and provided a blood sample for Proclarix testing. Importantly, the decision to do a biopsy was not based on Proclarix or MRI results. Exclusion criteria included patients having a history of PCa, serious illness (such as dementia or severe cardiovascular disease), treatment with 5‐reductase inhibitors, or contraindication to undergoing an mpMRI.

### 2.3 Proclarix testing

Serum samples for Proclarix testing were prospectively collected prior to plan biopsies. Samples were stored at −80°C, and the Proclarix assay was performed in batch for the entire patient cohort at a centralized lab (Proteomedix AG, Switzerland). Aside from patient age, the laboratory performing the assay was blinded to all clinical parameters and biopsy results. Performance of the Proclarix assay and subsequent risk score calculation was performed according to the manufacturer's instruction for use.^14^ The cut‐off used to evaluate the clinical performance of Proclarix was set at 10%.

### 2.4 Prostate MRI and biopsy procedures

mpMRIs were conducted on a 3T MAGNETOM Skyra MRI system and used T2‐weighted, diffusion‐weighted and dynamic contrast‐enhanced sequences. All mpMRI exams were read by board‐certified radiologists using the Prostate Imaging‐Reporting and Data System (PI‐RADS) v.2.0 (2). Prostate volume was calculated in millilitres (mL) and was determined using the formula height × length × width × π/6.

Prostate biopsies were performed using the transperineal approach under general anaesthesia with the BiopSee platform (MedCom GmbH, Darmstadt, Germany). A saturation biopsy was performed on all patients with 1–3 biopsies cores obtained from each of the 20 modified Barzell zones.^26^ Additional targeted biopsy cores were taken of any suspicious lesion found on mpMRI (2–3 biopsy cores per lesion). Each collected biopsy core was individually reviewed by a board‐certified uropathologist, and in the case of PCa, a second pathologist verified the diagnosis and grade. csPCa was defined as grade group ≥ 2.

### 2.5 Statistical analysis

Sensitivity, specificity, positive predictive value (PPV) and negative predictive value (NPV) were calculated for the Proclarix test as well as PSA, PSA density and Proclarix density. For calculations of PSA and Proclarix density, prostate volume was determined by mpMRI. To compare the patient characteristics including results of the above mentioned parameters in the groups of patients with and without PCa, the Mann–Whitney *U* test was used for continuous variables and the Fisher's exact test for categorical variables. To assess differences between sensitivity and specificity, the McNemar test was used. *p*‐values for NPV and PPV were calculated according to Moskowitz and Pepe.^27^, ^28^ Receiver operating characteristic (ROC) curves were constructed, and areas under the curve (AUCs) were calculated by using the trapezoidal rule. The statistical analysis was carried out using SPSS version 28.0. (IBM Corp). *p*‐values < 0.05 were considered as statistically significant.

## 3 RESULTS

The baseline characteristics of the study cohort are summarized in Table 1. A total of 65 (43%) was diagnosed with csPCa. Grade group 2 PCa was found in 27 (18%), grade group 3 in 16 (10%), grade group 4 in 18 (12%) and grade group 5 in 4 (3%) of the patients. Age, volume, PSAD, Proclarix and Proclarix density did significantly differ between patients with and without csPCa (*p* < 0.001 for all comparisons), whereas tPSA was not significantly different (*p* = 0.227).

**TABLE 1:** Patient characteristics.

Characteristics	Total	No cancer or ciPCa	csPCa	*p*‐value
Patients, *n* (%)	150 (100)	85 (57)^a^	65 (43)	‐
Age (year)	65 (60–71)	62 (59–68)	68 (63–73)	<0.001
tPSA (ng/mL)	5.8 (3.7–9.8)	4.9 (3.1–7.8)	7.5 (4.3–11.5)	0.227
Volume (mL)	45.9 (34.8–64.9)	50.1 (38.5–69.8)	41.8 (29.0–56.6)	0.003
PSA density (ng/mL^2^)	0.12 (0.08–0.20)	0.10 (0.07–0.14)	0.17 (0.12–0.29)	<0.001
Proclarix score	30.8 (16.9–46.0)	23.9 (14.7–36.7)	40.4 (21.3–58.4)	<0.001
Proclarix density (%/mL)	0.44 (0.25–0.71)	0.90 (0.57–1.45)	0.59 (0.31–1.03)	<0.001

*Note*: Data presented in median and interquartile ranges (IQR).

Abbreviations: ciPCa, grade group < 2; csPCa, clinically significant prostate cancer (defined as grade group ≥ 2); tPSA, total prostate specific antigen.

^a^ 63 (42%) pts no cancer, 22 (15%) pts ciPCa.

**FIGURE 1:**
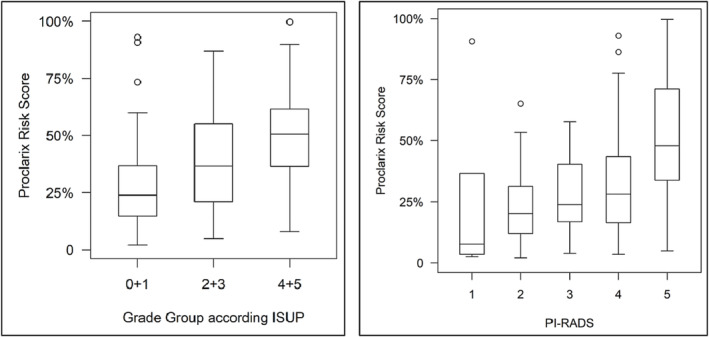
Proclarix risk score (0%–100%) in association with grade group according to ISUP (left) and with PI‐RADS (right).

The Proclarix risk score (score range from 0% to 100%) was significantly associated with higher PI‐RADS score and cancer grade (for both *p* < 0.001) (Figure 1). When applying a risk score cut‐off of 10%, the Proclarix test was found to have a sensitivity of 94% (95%CI: 88%–100%) and a specificity of 19% (95%CI: 11%–27%) for the diagnosis of csPCa. Values of NPV and PPV were found to be of 80% (95%CI: 62%–98%) and 47% (95%CI: 38%–56%), respectively (Table 2). Using this cut‐off to determine the need for a prostate biopsy, the Proclarix test would avoided the need for a prostate biopsy in 16 patients (11%), with four (6%) of cases of csPCa being missed. This included three patients with grade group 2 PCa and one with grade group 4 disease.

**TABLE 2:** Performance of Proclarix and PSA density across the whole cohort.

Parameter	Proclarix	PSA density	*p*‐value
Cut‐off	10	0.15	‐
Sensitivity, %	94 (88–100)	58 (46–70)	<0.001
Specificity, %	19 (11–27)	82 (74–90)	<0.001
NPV, %	80 (62–98)	72 (63–81)	0.343
PPV, %	47 (38–56)	72 (60–84)	<0.001

Compared to Proclarix, PSAD with a cut‐off of 0.15 ng/mL^2^ demonstrated a sensitivity of 58% (95%CI: 46%–70%) and a specificity of 82% (95%CI: 74%–90%). The PPV was 72% (95%CI: 60%–84%), and NPV was 72% (95%CI: 63%–81%). Based on these data, PSAD would have avoided 70 (47%) patients of unnecessary biopsies, with 27 out of 65 (42%) patients with csPCa being missed, including seven patients with grade group 5 PCa and seven patients with a grade group 4 cancer.

**FIGURE 2:**
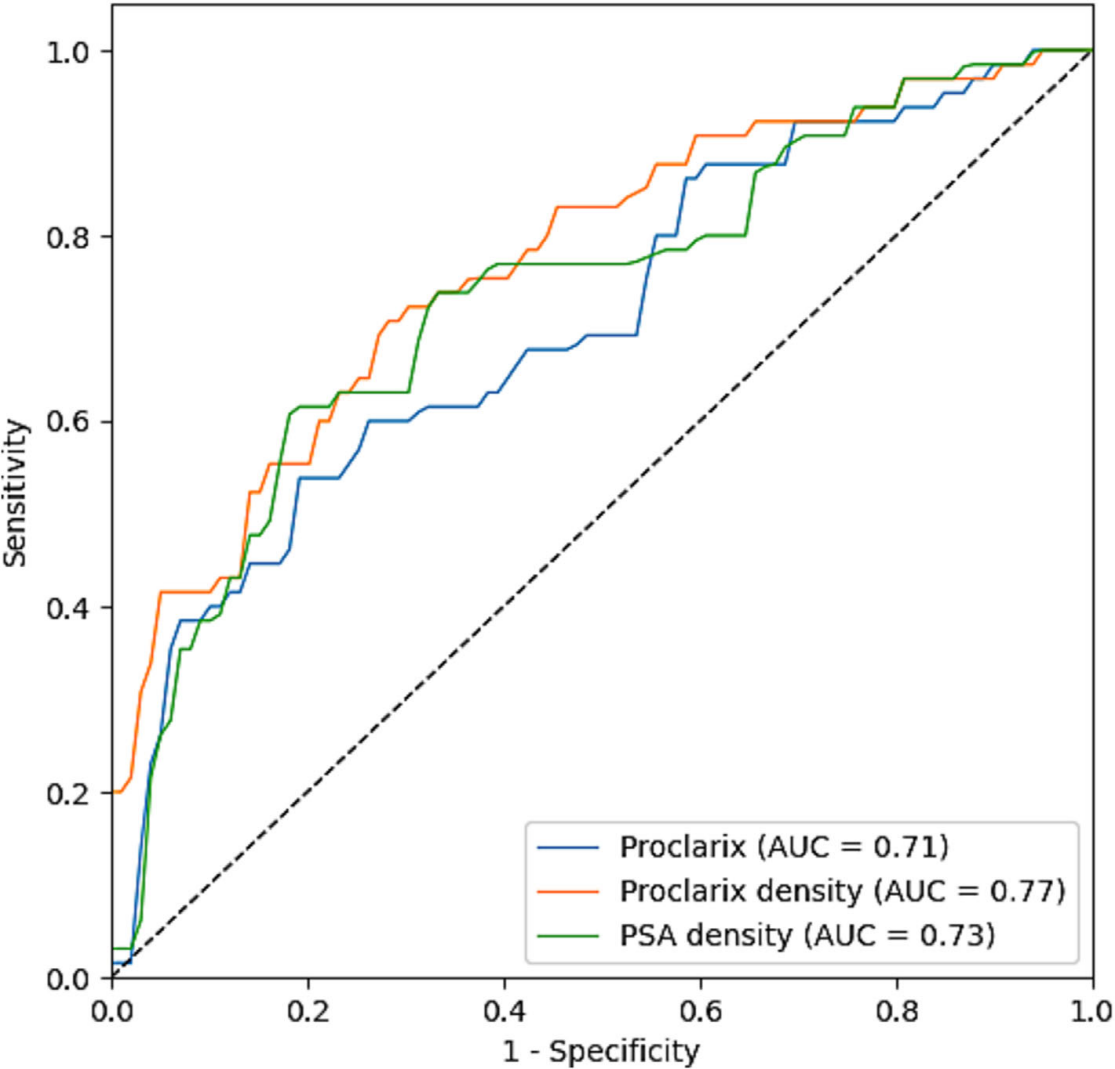
ROC curves for Proclarix, PSA density and Proclarix density with AUC of 0.71, 0.73 and 0.77, respectively.

To test discrimination for csPCa, ROC curves for Proclarix, PSAD and Proclarix density for the whole cohort were applied (Figure 2). Proclarix density showed the highest level of discrimination, with an area under the curve (AUC) of 0.77 (95%CI: 0.69–0.85), compared to Proclarix (AUC of 0.71; 95%CI: 0.62–0.79) and PSA density (AUC of 0.73; 95%CI: 0.65–0.82).

The performance of Proclarix, PSAD and Proclarix density was evaluated in a subgroup of men with indeterminate PI‐RADS 3 lesion seen on mpMRI (*n* = 28). Six out of 28 (21%) men in this subgroup were diagnosed with csPCa, whereas the other 22 (79%) had either a negative biopsy or grade group 1 PCa. In this subgroup of men, the Proclarix test would have avoided three (11%), without missing any of the six cases of csPCa. In contrast, PSAD would have avoided 18 biopsies (64%), while missing four cases of csPCa. When Proclarix density was compared with PSAD using optimized cut‐offs to yield similar sensitivity of 100% each, Proclarix density showed a specificity of 41% (95%CI: 20–61), while PSAD demonstrated a specificity of 14% (95%CI: 0–28, *p* = 0.03).

**FIGURE 3:**
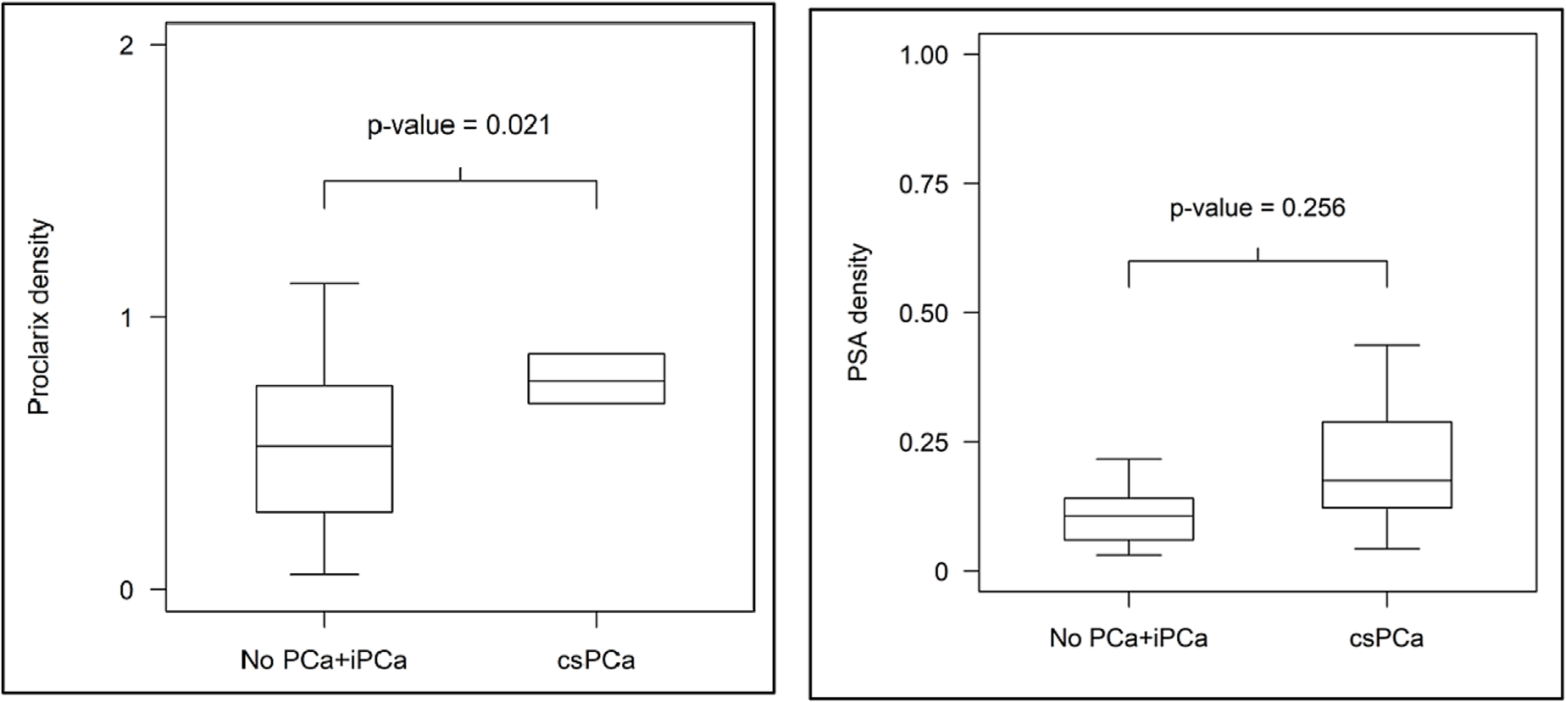
Boxplots show discrimination of no PCa and iPCa versus csPCa in patients with PI‐RADS 3 findings by Proclarix density (left) and PSA density (right).

Furthermore, Proclarix density could significantly discriminate ciPCa/noPCa from csPCa in the PI‐RADS 3 subpopulation (*p*‐values of 0.021 for Proclarix density and 0.256 for PSAD, respectively) (Figure 3). Results of this analysis are summarized in Table 3.

**TABLE 3:** Comparison of the clinical performance for the detection of csPCa of Proclarix, PSAD and Proclarix density in the patient population with PI‐RADS 3 findings (*n* = 28).

Parameter	Proclarix	PSA density	*p*‐value^a^	Proclarix density	PSA density	*p*‐value^b^
Cut‐off	10	0.15	‐	0.395^c^	0.043^c^	‐
Sensitivity, %	100 (100–100)	67 (29–100)	0.157	100 (100–100)	100 (100–100)	1.000
Specificity, %	14 (0–24)	82 (66–98)	<0.001	41 (20–61)	14 (0–28)	0.034
NPV, %	100 (100–100)	90 (77–100)	0.157	100 (100–100)	100 (100–100)	1.000
PPV, %	24 (7–41)	50 (15–85)	0.012	32 (11–52)	24 (7–41)	0.034

^a^ Comparison between Proclarix and PSA density.

^b^ Comparison between Proclarix density and PSA density at optimal cut‐offs.

^c^ Optimized cut‐offs for this patient population with PI‐RADS 3 findings.

## 4 DISCUSSION

The purpose of this study was to assess the performance of Proclarix, a novel blood‐based biomarker for the detection of csPCa, in a contemporary cohort of men who underwent an mpMRI followed by saturation biopsy of the prostate. In all cases, the decision to perform a biopsy was made independent of the results of the mpMRI or Proclarix test results, allowing for an unbiased assessment of the diagnostic accuracy of this novel biomarker against a highly rigorous truth standard. In our study, Proclarix demonstrated a sensitivity of 94% and a NPV of 80% for identifying csPCa using a 10% risk score cut‐off, missing four out of 65 csPCa cases. The specificity of 19% was rather low, translating in only 16 out of 85 unnecessary biopsies that could have been avoided. Compared to PSA density and Proclarix alone, Proclarix density showed the highest level of discrimination with an AUC of 0.77. In the subgroup of men with indeterminate PI‐RADS 3 lesions, Proclarix achieved a sensitivity and NPV of 100%, outperforming PSAD, while its specificity at 14% was again relatively low.

Our study confirms Proclarix's high sensitivity for csPCa and shows the correlation with the results of mpMRI. The results were similar to those previously published in other studies.^15^, ^17^, ^18^, ^29^ When the manufacturer‐recommended cut‐off of 10% was applied, Proclarix would have missed only 6% of csPCa, while PSAD would have missed 41% csPCa. These results are consistent with recently published data by Campistol et al.,^19^ where Proclarix sensitivity outperformed PSAD by missing only 6, respectively, 23 out of 232 csPCa patients. However, Proclarix showed only moderate performance for the avoidance of unnecessary biopsies of men harbouring either only ciPCa or no cancer (specificity of 19%), meaning only 16 out of 85 unnecessary biopsies could have been avoided. However, by including prostate volume in the Proclarix test as Proclarix density, we could show the highest discriminatory power for csPCa compared to PSAD or Proclarix alone, with an AUC of 0.77. These results are in line with a recent study by Steuber et al.^15^ on 121 mpMRI‐fusion biopsies. The study found that the specificity increased from 22% when using Proclarix alone to 33% with Proclarix density, while PSAD showed a significantly lower specificity of only 8% (*p* < 0.001). By utilizing Proclarix density, unnecessary biopsies could be avoided in up to one‐third of cases.

Men with indeterminate PI‐RADS 3 findings might often be candidates for continued PSA surveillance rather than biopsy, but appropriate selection for biopsy avoidance in these subpopulation is challenging. Hence, we performed a sensitivity analysis for Proclarix performance in this specific group and found Proclarix density with a specific cut‐off of 0.395 to maintain optimal sensitivity but gaining relevant specificity of 41% compared to PSAD (only 14% specificity). Therefore, Proclarix density could potentially reduce the need for unnecessary prostate biopsies in this subgroup, while still allowing for sufficient detection of csPCa.

Our suggested diagnostic pathway would be to use Proclarix density when the volume of the prostate can be established either by abdominal or transrectal ultrasound. A prostate volume estimation from a DRE is not recommended. When dealing with a PI‐RADS 3 lesion, the volume should ideally be calculated from MRI data.

Despite these promising results, our study is not without limitations. Most relevant is the relative small population size and the restriction of testing to one Western European clinical site with mostly Caucasian patients. Furthermore, our study did not include a validation cohort, limiting the generalizability of our findings.

## 5 CONCLUSION

Proclarix shows good performance in detecting csPCa but is hampered by a relatively low specificity, which would still result in many unnecessary biopsies. Proclarix density outperformed Proclarix alone for csPCa detection. In the subgroup of men with PI‐RADS 3 lesions on mpMRI, Proclarix density showed very good accuracy in detecting all csPCa cases while saving up to 40% of unnecessary biopsies.

## AUTHOR CONTRIBUTIONS

*Conceptualization*: Basil Kaufmann and Cédric Poyet. *Data collection*: Sharon Fischer. *Data analysis*: Basil Kaufmann. *Writing—original draft*: Basil Kaufmann and Cédric Poyet. *Writing—review and editing*: all authors. All authors have read and agreed to the published version of the manuscript.

## CONFLICT OF INTEREST STATEMENT

A. A. and R. S. received/held stock options and salaries and founder shares (R. S.) of Proteomedix AG. A. A. and R. S. are inventors of the following patent application (WO2018011212) as well as R. S. on patent application (WO2009138392). All other authors declare no conflicts of interest.

## Data Availability

The data presented in this study are available on request from the corresponding author.
